# An Energy-Efficient Sensing Matrix for Wireless Multimedia Sensor Networks

**DOI:** 10.3390/s23104843

**Published:** 2023-05-17

**Authors:** Vusi Skosana, Adnan Abu-Mahfouz

**Affiliations:** 1 Department of Electrical, Electronic and Computer Engineering, University of Pretoria, Pretoria 0028, South Africa; a.abumahfouz@ieee.org; 2 Council for Scientific and Industrial Research, Pretoria 0001, South Africa

**Keywords:** chaotic sequences, energy efficiency, image quality, sensing matrix, wireless multimedia sensor network, wireless sensor network

## Abstract

A measurement matrix is essential to compressed sensing frameworks. The measurement matrix can establish the fidelity of a compressed signal, reduce the sampling rate demand, and enhance the stability and performance of the recovery algorithm. Choosing a suitable measurement matrix for Wireless Multimedia Sensor Networks (WMSNs) is demanding because there is a sensitive weighing of energy efficiency against image quality that must be performed. Many measurement matrices have been proposed to deliver low computational complexity or high image quality, but only some have achieved both, and even fewer have been proven beyond doubt. A Deterministic Partial Canonical Identity (DPCI) matrix is proposed that has the lowest sensing complexity of the leading energy-efficient sensing matrices while offering better image quality than the Gaussian measurement matrix. The simplest sensing matrix is the basis of the proposed matrix, where random numbers were replaced with a chaotic sequence, and the random permutation was replaced with random sample positions. The novel construction significantly reduces the computational complexity as well time complexity of the sensing matrix. The DPCI has lower recovery accuracy than other deterministic measurement matrices such as the Binary Permuted Block Diagonal (BPBD) and Deterministic Binary Block Diagonal (DBBD) but offers a lower construction cost than the BPBD and lower sensing cost than the DBBD. This matrix offers the best balance between energy efficiency and image quality for energy-sensitive applications.

## 1. Introduction

A Wireless Multimedia Sensor Network (WMSN) consists of optical sensor nodes deployed to an area of interest and at least one data sink in different topologies [1]. The nodes study various areas and send data to at least one sink. Multi-hop routing can be part of the link, raising the requirement of a high compression ratio.

These WMSNs have to function in energy-constrained environments that demand novel compression schemes to lessen the bandwidth utilisation and computational complexity [2]. Energy conservation is one of the three energy management methods exploited in Wireless Sensor Networks (WSN); the others are energy transfer and energy harvesting [3]. However, the large data transfers for WMSN make energy conservation the greatest tool. Compressed Sensing (CS) was introduced by Pudlewski et al. [4] as a means to overcome this. The CS mechanism is appropriate for WMSN owing to its low complexity, high compression rate, and robustness to transmission errors [5].

Even though there have been other approaches to complexity reduction, such as sparsity transforms [5,6], they come at an intractable price of image quality [7].

Traditionally, each CS measurement maps an image onto an unrepeated measurement vector [8]. However, this imposes a sizable memory footprint when the signal is significant, such as in high-resolution images. In [9], Gan introduced Block-Compressed Sensing (BCS) to break up the image into blocks and use the same measurement vector for every block, thus vastly minimizing the footprint.

Bajwa et al. [10] significantly reduced sensing and storage complexity by proving the efficacy of Toeplitz and circulant sensing matrices. With these, just the first row needs random entries, while the other rows are transformations of the first. These matrices are called semi-deterministic, and matrices with no random entries are fully deterministic.

Some researchers have been looking at optimisation-based matrices that minimise the mutual coherence with the sparsity transform [11,12,13,14,15,16], but these matrices cannot be constructed on WMSN nodes. More recent approaches have been training the sensing matrix based on a signal dataset [17,18,19], but similarly, this is not ideal for WMSN nodes.

Arjoune et al. [20] contrasted different types of sensing matrices with great detail. They investigated using experiments on 1D signals on a central processing unit (CPU)-powered computer. However, these time measurements (and possible energy consumption) are not characteristic of WMSNs using microcontroller units (MCUs).

Determinsistic sensing matrices have outperformed dense ones in many studies [21,22,23,24,25,26,27,28,29,30]. Semi-deterministic matrices are being displaced in popularity by fully deterministic ones, particularly matrices based on chaotic sequences [24,27,28].

### 1.1. Motivation

Sensing complexity has yet to be analysed or compared with previous work by most researchers. Storage complexity has been studied more, but it is not a good proxy for sensing complexity. Energy consumption has been studied even less. A sensing matrix is needed that can meet the need for energy efficiency and recovery performance and compare favourably with current approaches.

### 1.2. Contribution and Organisation

In this paper, we analyse, evaluate, and compared the leading deterministic measurement matrices. We propose a new measurement matrix that addresses the identified shortcomings and compare it with the leading matrices in terms of its energy efficiency for construction and sensing as well as recovery performance.

- We review the leading deterministic matrices regarding image quality and computational complexity.
- We identify the best techniques for designing deterministic sensing matrices.
- We evaluate the effect of random numbers and block size on recovery performance and energy consumption.
- We propose a sensing matrix based on random sample positions.

In Section 2, research output on Sensing Matrices is reviewed. The theoretical background is introduced in Section 3. In Section 4, the new sensing matrix is proposed. Experiments are detailed in Section 5, and the results are presented in Section 6. The results are discussed in Section 7, and the conclusion is presented in Section 8.

## 2. Related Work

There has been considerable work on developing sensing matrices for addressing the computational complexity and reconstruction quality of CS. In the next subsections, the current semi-deterministic and fully deterministic approaches are reviewed. Amongst semi-deterministic matrices, the Binary Permuted Block Diagonal (BPBD) had the most promise because of its high sparsity. The other matrices had lower sparsity and complex computations. The Deterministic Binary Block Diagonal matrix (DBBD) had the highest sparsity amongst fully deterministic matrices, which is based on the BPBD. The semi-deterministic matrices tend to have complex computations, while the fully deterministic matrices have low sparsity.

### 2.1. Semi-Deterministic Matrices

He et al. [31] proposed the BPBD to address hardware implementation and sensing efficiency. The matrix is binary and significantly sparse, which substantially simplifies hardware implementation and reduces sensing computations. This matrix requires random numbers to permute the columns during construction, which adds complexity depending on the source of the random numbers. The researchers contrasted the BPBD with other matrices with experiments on MATLAB. They found that their measurement matrix gives comparable recovery performance to scrambled block Hadamard, scrambled Fourier, partial Noiselet, and Gaussian and that their measurement matrix outperforms the other matrices at a sampling rate of 10%.

In [23], two memory-efficient measurement matrices were proposed. The first one is called the Combination matrix, and it is the Kronecker product of the Gaussian and Toeplitz measurement matrices. This approach reduces the effective number of required random entries, but the multiplication increases the construction complexity. The second matrix is called the Hybrid matrix, and it is generated through a compound of Toeplitz and Binary matrices. The authors evaluated their matrices using experiments on the TelosB platform. They observed that their matrices performed better than the Gaussian matrix based on energy consumption and recovery performance. The Hybrid performed better than the Combination matrix.

A combination of Toeplitz, Hankel, and circulant matrices was proposed in [26]. First, the Toeplitz matrix is constructed from Gaussian random entries. The Hankel and circulant matrices are constructed from transformations of the Toeplitz matrix. Finally, the sensing matrix is constructed by summing all three matrices. These researchers compared their matrix to the Gaussian matrix on the TelosB platform. The authors found that their matrix outperformed the Gaussian matrix on recovery performance and energy consumption.

### 2.2. Fully Deterministic Matrices

Ravelomanantsoa et al. [30] proposed a simple deterministic measurement matrix that facilitates hardware implementation. This sensing matrix is named the DBBD and simplifies the BPBD by eliminating random permutations. The authors tested their proposed measurement matrix on a 1D electrocardiogram (ECG) and an electromyogram (EMG) on an MSP-EXP430G2 LaunchPad development board. They experimented to assess their proposed matrix against the Gaussian and BPBD matrices. They exploited the DCT and their proposed OMP-variant reconstruction algorithm. Their measurement matrix performed better than both the Gaussian and BPBD in terms of recovery accuracy measured in SNR and computational complexity.

In [24], the authors exploited the simplicity of the Logistic mapping chaotic systems to develop the incoherent rotated chaotic (IRC) sensing matrix. They ignored the first 1000 elements of the sequence and downsampled the subsequent elements using an interval of *d* to improve the randomness. This means that to generate *t* sampled values, a chaotic sequence of length l=1000+dt must be generated. The IRC matrix requires only *n* factors that are rotated for each row, which improves storage compared with other chaotic matrices. The authors added an incoherence factor η, which is multiplied for each rotation and can be separated into a matrix ω. However, the multiple multiplications from the incoherence factor add computational complexity to the generation process. The matrix was compared to the Gaussian matrix using MATLAB experiments. The authors found that their matrix outperforms the Gaussian one, but it is a challenge to select the incoherence factor, which has a meaningful impact on the reconstruction performance.

Gan et al. [27] presented a measurement matrix that exploits bipolar chaotic sequences to simplify storage and multiplication. However, the sequences are generated from the Chebyshev chaotic system that entails the cos function, which is expensive to implement in MCU. A threshold value is applied to the sequence to make a bipolar matrix. The researchers contrasted their matrix to the Gaussian, Bernoulli, improved Hadamard, and dense Chebyshev matrices through experiments. Their matrix performed comparably to the other matrices and performed the best at low sampling rates.

Sun et al. [28] presented the Chaos-Bernoulli Block Circulant Matrix to conserve transmission capacity. They produced a pseudo-random sequence by choosing the initial value and sampling interval. The sequence is derived from a nonlinear Hybrid Chaotic map where the Logistic map and Tent map are combined. The authors ignored the first 1000 values of the sequence to improve the randomness. They also sampled the chaotic sequence at an interval to improve the independence of the sampled values. The sampled sequence is then mapped through the sign function. The authors built a block-circulant matrix from a chaotic sequence to reduce the storage and implementation requirements. The final measurement matrix was obtained by randomly selecting *M* rows from the block circulant matrix. The matrix was compared to the Gaussian, Bernoulli, Hybrid chaotic, and Gaussian circulant matrices using numerical experiments. The authors found that their matrix outperformed the other matrices.

### 2.3. Summary

The relative performance of the different matrices is presented in Table 1. The complexity was assigned values of Low, Medium, or High. The reconstruction accuracy was judged based on whether the matrix had higher image quality than the Gaussian matrix.

## 3. Preliminaries

This section gives a short background on the compressive sensing and WSN used for the development of the proposed scheme.

### 3.1. Compressed Sensing

The CS framework allows for the accurate recovery of data captured in a vector of length *N* from only M≪N samples. CS depends on sparsity and incoherence to ensure signal recovery [32]. Sparsity is the quality of how few non-zero entries belong to a vector representing the signal. A signal can also be made sparse by applying a transformation, Ψ, to a domain where the signal can be sparse. Incoherence is the quality of low correlation between the sparsifying transform and the sensing matrix, Φ [32]. The incoherence can be measured through mutual coherence, shown in Equation (1), where lower values mean more incoherence.

(1) $$\mu \left(\Phi ,\Psi^{-1}\right)=\sqrt{N}\max_{i\neq j}\frac{|\phi_{i}^{T}\psi_{j}^{-1}|}{{∥\phi_{i}∥}_{2}{∥\psi_{j}^{-1}∥}_{2}},$$

The mutual coherence is the maximum normalised similarity between the row vectors of Φ, $\phi_{i}$ and the column vectors of Ψ, $\psi_{j}$.

Once the signal has been compressively sensed in *M* measurements, it can be recovered in its entirety using numerical optimisation algorithms. The algorithms attempt to recover an unknown signal *Z* from a few measurements. The signal to be compressed $x\in R^{N}$ can be expressed as

(2) x=Ψs,

where Ψ is the sparsity transform and *s* is the *N* length vector of the captured signal. The signal *x* is sparse, with a sparsity of *K*. When the measurement matrix is applied to *x*, we obtain a vector *y*.

(3) y=Φx=ΦΨs=Θs,

The robustness of CS depends on the restricted isometry property (RIP) of the sensing matrix. The RIP requires columns of Φ to be close to orthogonal. Random sensing matrices meet the RIP criteria with high probability on the condition that Equation (4) [32] holds. The RIP is also important in ensuring that CS can gracefully deal with additive noise.

(4) $$M\geq CK\log\left(\frac{N}{K}\right),$$

### 3.2. Peak Signal-to-Noise Ratio

Image quality is measured using the Peak-Signal-to Noise Ratio (PSNR). This metric has been shown to be robust at low sampling rates and high distortion [33]. This was a popular objective measure of image quality. PSNR is measured in decibels (dB) and raises in value with image quality; Equation 5 is the mathematical formulation [34].

(5) $$\begin{array}{cc} MSE= & \frac{1}{MN}\sum_{i=0}^{M-1}\sum_{j=0}^{N-1}{\left(\rho_{r}\left(i,j\right)-\rho_{p}\left(i,j\right)\right)}^{2}\\ PSNR= & 10\log\left(\frac{L^{2}}{MSE}\right) \end{array}$$

The PSNR has an opposite relationship to the mean square error (MSE), which is the cumulative squared error between the reference $\rho_{r}\left(i,j\right)$ and the processed image $\rho_{p}\left(i,j\right)$, and *L* is the dynamic range of the pixel value.

### 3.3. Wireless Multimedia Sensor Networks

There have been many WMSN node designs, ranging from 8 MHz using the ATmega128L to 624 MHz using the XScale PXA270. In [35], the authors classify these WMSN nodes into Low and High performance. The microcontroller unit (MCU) is an integral part of the sensor node and is responsible for a significant amount of its total energy consumption [36]. The focus of this study will be on low-performance nodes, in particular, the TelosB mote, for energy analysis. This mote has a Texas Instrument MSP430 MCU that runs at 8 MHz with 10 KB of RAM and 1 MB of an external flash. The mote has two AA batteries, each with a capacity of around 2850 mAh.

The choice of sensing matrix design affects the required number of random numbers for generating the measurement matrix, as well as the number of matrix multiplications during sensing. The Bernoulli matrix with ±1 entries was favoured for ease of implementation [37] by eliminating the multiplications. Another popular matrix is the Binary Sparse Random matrix, which has a small *d* number of non-zero entries per column, thus eliminating most addition operations [38].

Two choices exist for obtaining random entries for a measurement matrix, retrieval from memory or random generation. Retrieval from memory has the drawback of requiring large storage space, and non-volatile memory requires significant energy to operate [39]. The random generation of measurement matrix columns has considerable energy consumption from complex mathematical functions such as log or sqrt [38]. Deterministic measurement matrices have been identified as a means to ease hardware requirements by obviating random number generation and/or large memory storage [30].

#### Energy Consumption

It has been observed that the current drawn by 8- to 32-bit MCUs deviates very slightly during active operation [40]. It is, therefore, sufficient to measure the time length of an operation to estimate relative energy consumption, as seen in Equation (6).

(6) $$E_{con}=V_{cc}\times I_{active}\times t_{op},$$

The time length $t_{op}$ can be estimated using the clock frequency *f* of the MCU and the number of instruction cycles required for the operation $N_{ic}$, as shown in Equation (7).

(7) $$t_{op}=\frac{N_{ic}}{f},$$

The MSP430 does not have a hardware multiplier, and thus, the implementation of complex mathematical operations requires multiple instruction cycles. In [41], the authors discuss efficient algorithms to implement multiplication and division on the MCU. In Table 2, the instruction cycle cost of different arithmetic and logical operations on the MSP430 MCU are listed [41,42]. In the table, each mathematical operation is listed and the instruction cycle cost is presented. All the arithmetic and logical operations require one instruction cycle except multiply and divide. These arithmetic operations require 29 and 22 cycles, respectively.

## 4. Proposed Sensing Matrix

In this section, an energy-efficient and high-fidelity sensing matrix for WMSN is proposed. Fully deterministic matrices commonly use chaotic sequences to replace random numbers, which give similar sparsity to the Bernoulli matrix. Another successful fully deterministic matrix is the DBBD, which has a static matrix but still requires N−M addition operations during sensing. Semi-deterministic matrices have had good sparsity, with the PCI requiring no add operations during sensing. The PCI also has low mutual coherence with most sparsity transforms [43]. The weakness of the PCI matrix is that it requires expensive random numbers, which is similar to other semi-deterministic matrices. This shortcoming is addressed by replacing the random numbers with a chaotic sequence. The construction complexity is further improved by replacing random permutation with random sample positions, which also reduces time complexity.

### 4.1. Deterministic Partial Canonical Identity Matrix

The PCI matrix is traditionally constructed by randomly choosing *M* rows from an N×N identity matrix. The PCI has been used in [43,44,45], but in these studies, the matrix computational complexity and recovery performance have not been investigated. The construction of the PCI is where the energy consumption, and recovery performance are determined.

A popular approach to improving the recovery performance of measurement matrices is the minimisation of mutual coherence with a sparsity basis of Ψ [11,13,14,15,16]. However, for the DPCI, mutual coherence is a constant, $\sqrt{2}$ [43].

### 4.2. Random Number Generation

Random number generation is an important factor in reducing computational complexity and recovery performance. Different random-number-generation algorithms were evaluated to find the most suitable ones in terms of simplicity and recovery performance.

#### 4.2.1. Linear Congruent Generator

The Linear Congruential Generator (LCG) is one of the best-known and oldest algorithms for generating pseudo-random numbers. This generator is simple to implement and has low storage complexity. The algorithm exploits recurrence to generate random numbers shown in Equation (8).

(8) $$\begin{array}{cc} X_{n+1}=\; & \left(aX_{n}+c\right)\;\mathrm{mod}\;m\\ x_{n}=\; & \frac{X_{n}}{m} \end{array}$$

Here, *X* is the state of the algorithm, and *x* is a random number between 0 and 1.0. The variables *a*, *c*, and *m* are the multiplier, increment, and modulus, respectively. The choice of values for these variables determines the performance of the algorithm. The values used were *a* = 16,807, c=0, and *m* = 2,147,483,647 from [46]. The resulting energy consumption is shown in Equation (9) in terms of instruction cycles.

(9) $$\epsilon_{LCG}=\left(\epsilon_{mul}+2\epsilon_{div}\right),$$

#### 4.2.2. Logistic Map

The Logistic map is one of the simplest chaotic systems. This system is a suitable random number generator [47]. The equation of the system is shown in Equation (10). The μ is the system parameter, and each value of *n* is $x_{n}\in \left[0,1\right]$. A system parameter of μ=4.0 and an initial state of $x_{0}=0.4$ were used. The energy consumption for each number in the sequence is formulated in Equation (11) and measured in instruction cycles.

(10) $$\begin{array}{c} x_{n+1}=\mu x_{n}\left(1-x_{n}\right) \end{array}$$

(11) $$\epsilon_{logistic}=\left(\epsilon_{sub}+2\epsilon_{mul}\right),$$

#### 4.2.3. Tent Map

The Tent map is an iterative piece-wise linear difference equation with chaotic behaviour and a tent-like graph. This map is computationally efficient and has a uniform distribution of random numbers [48]. The equation is shown in Equation (12). The μ is the system parameter, and each value of *n* is $x_{n}\in \left[0,1\right]$. The system parameter μ=1.99 and initial state $x_{0}=0.4$ were used. The energy consumption for each number in the sequence is formulated in Equation (13) in instruction cycles.

(12) $$\begin{array}{cc} x_{n+1}=\; & \left\{\begin{array}{cc} \mu x_{n}, & \mathrm{if}\;0\leq x_{n}<\frac{1}{2}\\ \mu (1-x_{n}), & \mathrm{if}\;\frac{1}{2}\leq x_{n}\leq 1 \end{array}\right. \end{array}$$

(13) $$\epsilon_{tent}\approx \left(\epsilon_{comp}+\frac{1}{2}\epsilon_{sub}+\epsilon_{mul}\right),$$

### 4.3. Complexity Optimisation

The computational complexity of constructing the PCI was optimised by first exploiting deterministic random numbers and also by replacing random permutation with random sample positions. This optimisation reduced the computational and time complexity.

#### 4.3.1. Random Permutation

The Fisher–Yates Shuffle (FYS) algorithm was used to randomly permute the columns of the PCI matrix. The algorithm has two attractive properties: permutations are unbiased, and the shuffling has a linear time complexity O(n) [49]. The algorithm was used with values from the Tent map chaotic sequence as described in Algorithm 1. The energy consumption of each iteration of the algorithm is formulated using instruction cycles in Equation (14).

(14) $$\epsilon_{fys}=\left(N-1\right)\left(\epsilon_{tent}+\epsilon_{sub}+\epsilon_{mul}+\epsilon_{add}\right),$$

**Algorithm 1** Random Permutation.
1:**procedure** Shuffle(*A*)2:    $X_{0}$ = 0.4003:    *U* = 1.994:    **for** index *i* from N−1 to 1 in *A* **do**5:         **if** $X_{i-1}<0.5$ **then**6:               $X_{i}=UX_{i-1}$7:         **else**8:               $X_{i}=U\left(1-X_{i-1}\right)$9:         **end if**10:        *j* = (*i*− 1)$X_{i}$ + 111:        Swap A[i] and A[j]12:    **end for**13:    Return *A*14:
**end procedure**



#### 4.3.2. Random Sample Position

The fundamental characteristic of the PCI matrix is that there is only one non-zero entry per row. This fact is exploited to simplify the construction of the DPCI. The random sample position algorithm consists of generating a random column position for each row; see Algorithm 2.
**Algorithm 2** Random Sample Position.1:**procedure** Sample(*A*)2:     $X_{0}$ = 0.4003:     **for** row *i* from 1 to *M* in *A* **do**4:          $X_{i}=random\left(X_{i-1}\right)$5:          *j* = $N\times X_{i}$6:          A[i,j] = 1.07:     **end for**8:     Return *A*9:**end procedure**

The new construction maintains the orthogonal relationship between the columns, where the non-zero row entries do not coincide between columns. This arrangement maintains the RIP properties of the original shuffling mechanism. The new construction is more sensitive to the quality of the random number generator to distribute the non-zero entries evenly across columns. The randomness of the distribution will also be sensitive to the block size *N* through the sample size *M*.

The energy consumption of the algorithm is formulated in Equation (15) in instruction cycles. The permutation requires *M* iterations to approximate the shuffling of *N* columns. Each iteration entails only one complex computation, that of a Tent random number. This eliminates most of the computational complexity from shuffling. The algorithm also reduces the required random numbers and iterations from N−1 to *M*.

(15) $$\epsilon_{rsp}=M\epsilon_{tent},$$

**Figure 1:**
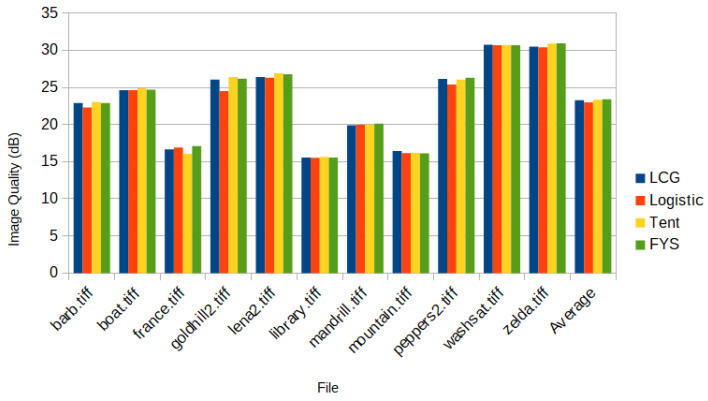
The recovery performance of different variants of DPCI.

**Figure 2:**
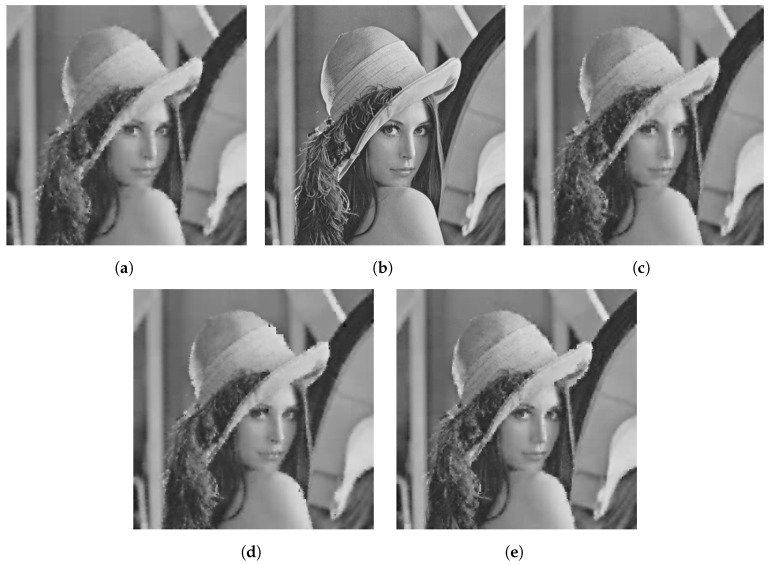
Lena2 from different variants of DPCI. (**a**) LCG. (**b**) Original. (**c**) FYS. (**d**) Logistic. (**e**) Tent.

This algorithm was tested with all three random number generators, LCG, Logistic, and Tent. Finally, it was compared with the traditional implementation using random permutation; see Figure 1 and Figure 2. The traditional implementation with FYS performed the best in terms of recovery quality, but the LCG and Tent had a competitive performance. The Tent implementation was chosen for further evaluation because of the lower computational complexity.

## 5. Experimental Evaluation

MATLAB was used for compressed sensing experiments on a Windows 10 computer with 16 GB RAM and an Intel Core i7 CPU. In order to make the results representative, many images were utilised during the experiments. A selection of images from the collection of greyscale images from the Waterloo Repertoire GreySet2 [50] was exploited in 512 × 512 sizes in order to assess image quality. The collection consists of 11 images of a wide range of subjects such as people, landscapes, animals, objects and posters (with text and sub-figures); the dataset is detailed in Table 3. The image was divided into blocks of 8 × 8, 16 × 16 and 32 × 32 pixels. Every block was made sparse using DCT and sampled using different sensing matrices. The images were recovered using block-based CS sampling and smoothed projected Landweber (BCS-SPL) method [51]. Experiments were conducted to assess each sensing matrix at a low sampling rate of 10% to evaluate the matrices at the most challenging and energy-efficient level. Each experiment was repeated multiple times to account for statistical variations using five trials. The recovery accuracy was measured using the average image quality in the form of PSNR. The energy consumption was evaluated using analytical methods.

## 6. Results

The results of the experiments are presented in this section. The recovery performance is first presented using image quality. Finally, the energy consumption is analysed.

### 6.1. Recovery Performance

The quantitative recovery performance of the different matrices is presented using image quality in Table 4, Table 5 and Table 6. These tables cover disparate block sizes, 8 × 8, 16 × 16, and 32 × 32. The PSNR for each recovered image is listed for each sensing matrix, and the average over all the images is listed on the last row of each table.

In Table 4, the highest image quality was from the Zelda file, with 32.19 dB compressed with the DBBD matrix. The lowest image quality was on the Library file, with 15.19 dB compressed using the DPCI matrix. The DBBD had the highest average performance at 24.42 dB, followed by the BPBD at 24.01 dB. The DPCI, at 22.94 dB, had the third best, and finally, the Gaussian matrix was the lowest at 21.84 dB.

Table 5 shows that the highest image quality from the Zelda file, with 33.36 dB compressed using the DBBD matrix. The lowest image quality was found in the Library file, with 15.65 dB compressed using both the DPCI and Gaussian matrices. The DBBD had the best average performance of 25.28 dB; the second was the BPBD with 24.03 dB. The Gaussian was third with 23.53 dB, and the DPCI had 23.35 dB.

Table 6 shows that the highest image quality was obtained from the Zelda file, with 32.71 dB compressed with the DBBD matrix. The lowest image quality was found in the Library file, with 14.91 dB compressed using the DPCI matrix. The DBBD had the highest average performance with 24.73 dB, followed by the BPBD with 23.96 dB. The Gaussian had the third-best performance with 22.94 dB, and the DPCI was last with 22.81 dB.

**Figure 3:**
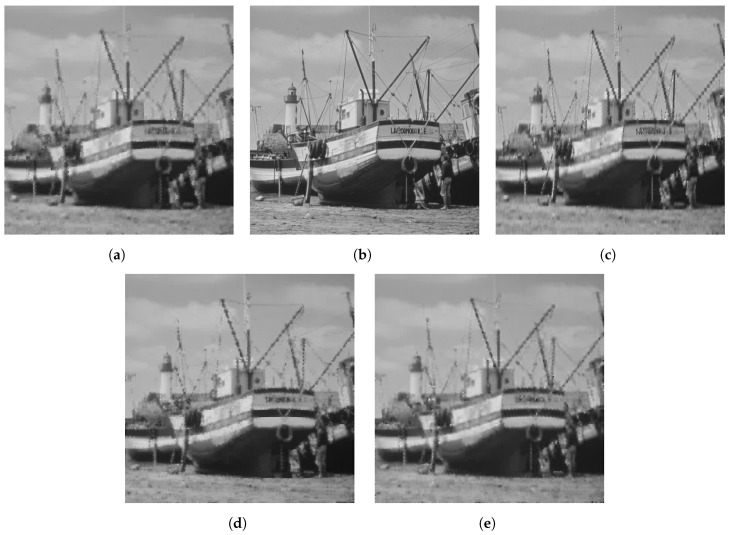
Reconstructed Boat images for block size 8. (**a**) BPBD. (**b**) Original. (**c**) DBBD. (**d**) DPCI (**e**) Gaussian.

**Figure 4:**
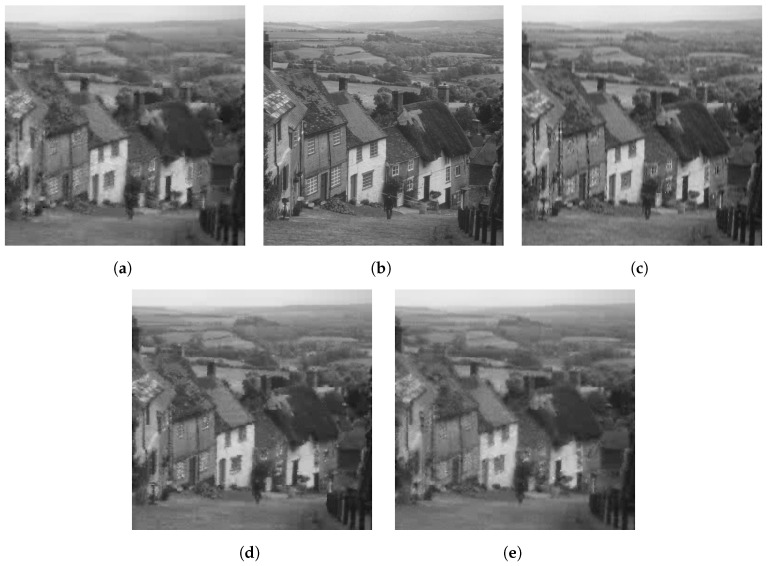
Reconstructed Goldhill2 images for block size 16. (**a**) BPBD. (**b**) Original. (**c**) DBBD. (**d**) DPCI. (**e**) Gaussian.

**Figure 5:**
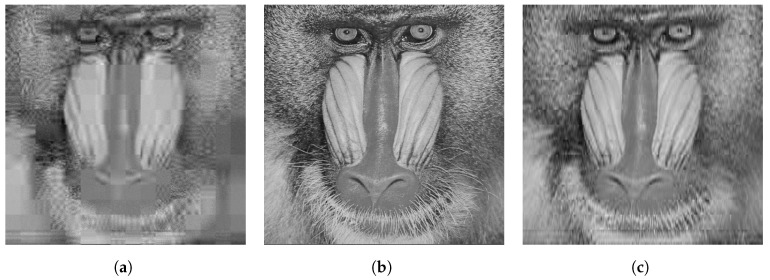

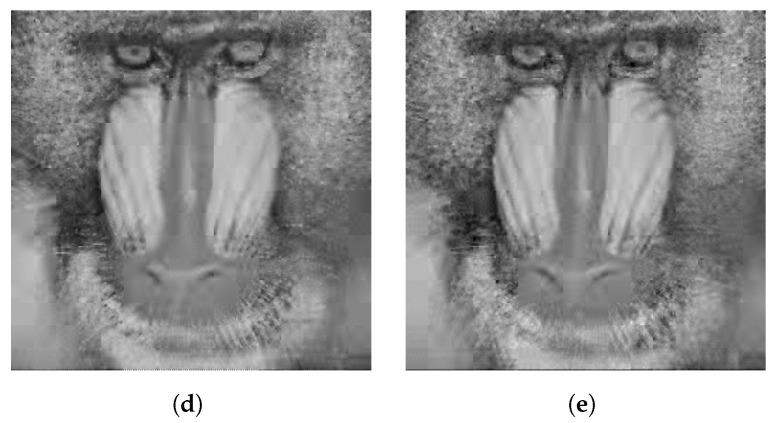
Reconstructed Mandrill images for block size 32. (**a**) BPBD. (**b**) Original. (**c**) DBBD. (**d**) DPCI (**e**) Gaussian.

The qualitative difference in performance of each of the sensing matrices is shown in Figure 3, Figure 4 and Figure 5 through a few examples. Four images that were compressed using the Gaussian, DBBD, BPBD. and DPCI are shown in each figure.

Figure 3 shows Boat images compressed using a block size of 8 × 8. In the figure, the DBBD had the sharpest detail but had significant pixelation artefacts. The DPCI had the second-best detail, where small details such as the ship rigging are visible. However, the DPCI had pixelation artefacts. The BPBD had the third-best detail, where sections of the ship rigging are still visible. The Gaussian had no pixelation artefacts but had the lowest detail. These results are corroborated quantitatively in Table 4.

Figure 4 shows the GoldHill2 images compressed using a block size of 16 × 16. The DBBD had the best visual detail but had some colour distortions. The BPBD and DPCI had very similar performances. The Gaussian had the least detail but had no distortions. These results are similar to the quantitative results in Table 5.

Figure 5 shows Mandrill images compressed using a block size of 32. The DBBD had the best visual detail but had visible lines across the image. The DPCI and Gaussian had similar performance with the second-most detail. The BPBD had blocking artefacts and had the worst performance. The results are reflected in Table 6, where the performance numbers are close, with only the DBBD standing out.

### 6.2. Energy Consumption

The energy consumption of the different measurement matrices was compared using the MSP430x1xx Family MCU as a reference. The most energy-efficient operating region of the MCU is 1MHz, with a voltage supply of 2.2 V. In the active mode, the MCU current draw is 220 μA. The instruction cycle cost of mathematical operations is translated into energy consumption in Table 7. Multiplication and division are the most expensive operations, while the other operations are the same and lower in energy consumption.

The construction of the DPCI requires *M* Tent sequence numbers. The DPCI uses no multiplications or additions during sensing, unlike the BPBD and DBBD, which use N−M additions. The construction and sensing costs are shown in Table 8 for block sizes 8, 16, and 32, all at a 10% sampling rate. The DPCI only consumes energy for construction, while the BPBD and DBBD only consume energy for sensing. The total energy consumed for sensing the image is unaffected by block size and depends only on the size of the image. In this case, it was 114.2 μJ.

## 7. Discussion

The images had different levels of image quality after recovery. What is interesting is that three images had much lower performance, france.tiff, library.tiff, and mountain.tiff. These three images had an image quality below 20 dB for all the sensing matrices and block sizes. The first image is a poster with text and a high-contrast background. The second image is another poster with three sub-figures, text, and a white background. The last image is of a mountain range with high contrast.

The DBBD had the best quantitative and qualitative performance for all the block sizes. The matrix suffered from some distortion but had the greatest detail in all the images. The BPBD had the second-highest quantitative performance for all the block sizes, but the qualitative performance was only sometimes second-best. The DPCI had the third-best quantitative results for the block size of 8 × 8. However, it had the worst performance for the bigger sizes. The DPCI had the second- and third-best qualitative performance for some of the images. The Gaussian had the least detail and worst qualitative performance for most of the images but had no distortions.

The block size had different effects on each sensing matrix. The Gaussian had the lowest performance at the block size of 8 × 8 but had improved performance for 16 × 16 and 32 × 32. The BPBD had similar recovery performance for all the block sizes, while the DBBD and DPCI had peak performance at 16 × 16. The DPCI had the worst quantitative performance at 32 × 32, and it performed particularly severely for challenging images, with a relative dip in performance of almost 1 dB compared to the Gaussian. This performance drop may be caused by the statistical properties of the distribution of the non-zero values being affected by the sample size, *N*, but this needs further study.

The DPCI consumes energy for construction, unlike the DBBD, but this energy can be reduced by decreasing the block size. The DPCI consumes no energy for sensing, unlike the other matrices. Sensing energy is important because it determines the operating energy consumption of the node, and depending on the spectral and spatial resolution of the sensor, as well as the type and duration of the events being monitored, these sensing costs can accumulate exponentially.

## 8. Conclusions

Chaotic sequences can be used to address the shortcomings of semi-deterministic matrices to make fully deterministic matrices that are both energy-efficient and high in fidelity. The DPCI improves the computational complexity of the PCI by first replacing the random numbers with chaotic sequences and, secondly, by replacing random permutations with random sample positions. The DPCI is outperformed by the DBBD and BPBD regarding recovery performance but offers a significant computational advantage. The poorer recovery accuracy of the DPCI might be because it is much more sparse and takes fewer samples but this needs further investigation. The DPCI offers an attractive trade-off between recovery performance and energy efficiency that energy-sensitive applications can exploit. Some images are challenging for sensing matrices and need further investigation. Future research should focus on improving the recovery performance of the DPCI by investigating the sample positions that lead to better performance using training on an image dataset. These results can then be used to generalise across datasets and develop a universal sensing matrix.

## Figures and Tables

**Table 1:** Sensing matrix comparison.

Ref.	Type	Construction Complexity	Sensing Complexity	Image Quality
[31]	Semi	High	Low	Good
[23]	Semi	High	High	Good
[23]	Semi	High	Medium	Good
[26]	Semi	High	High	Good
[30]	Full	Low	Low	Good
[24]	Full	Medium	High	Good
[27]	Full	Medium	Medium	Good
[28]	Full	High	High	Good

**Table 2:** No. of cycles for operations.

Operation	Number of Instruction Cycles
Add	1
Subtract	1
Multiply	29
Divide	22
Compare	1

**Table 3:** Dataset images.

File	Subject	Image Content
barb.tiff	A person is photographed sitting on the floor with their face and most of the body visible.	The image has a lot of fine and regular textures, from the carpet floor to tablecloths with checked patterns.
boat.tiff	A small grounded sailboat is captured with a person standing next to it.	The image contains very little textures, mostly the ground; however, the textures are irregular.
france.tiff	Travel poster with smooth background and some text.	The image has grey-level gradient in the background but has very few textures.
goldhill2.tiff	The image captures conjoined houses built on a hill, and there is a person walking down the hill.	The image has a lot of texture from the roofs, ground, and trees in the background.
lena2.tiff	This image is a portrait of a person at close range and mostly captures their face.	The image has a mostly smooth background, but there is some rough texture from the feathers on the hat worn by the subject.
library.tiff	This is a poster with three subfigures; each subfigure has people and both small and large text.	The image has a lot of fine details and structures.
mandrill.tiff	The image is a close-up of the face of a mandrill.	The image has fine textures from the fur of the mandrill.
mountain.tiff	The image is a landscape of a mountain range with a body of water also visible.	The image has very high contrast, smooth and textured areas, and intricate details.
peppers2.tiff	This is a close-up image of peppers.	There are no textures but there are smooth surfaces and modest contrast.
washsat.tiff	This is a satellite image of a city where there are structures formed by the streets.	The image has a lot of fine details but is largely dark.
zelda.tiff	This is a portrait of a person where the background is out of focus and the face dominates the frame.	The image consists largely of smooth surfaces and almost no textures.

**Table 4:** Image quality results for 8 × 8.

File	PSNR (dB)			
	Gaussian	BPBD	DBBD	DPCI
barb.tiff	21.62	23.40	23.47	22.63
boat.tiff	24.16	25.32	25.84	24.64
france.tiff	15.65	17.78	18.32	16.89
goldhill2.tiff	25.47	26.71	26.56	26.25
lena2.tiff	25.57	27.51	28.13	26.28
library.tiff	15.58	16.15	16.74	15.13
mandrill.tiff	19.80	20.57	20.67	19.79
mountain.tiff	15.87	16.97	17.44	16.10
peppers2.tiff	25.33	27.16	27.57	25.08
washsat.tiff	22.46	31.33	31.75	29.66
zelda.tiff	28.67	31.23	32.19	29.89
Average	21.84	24.01	24.42	22.94

**Table 5:** Image quality results for 16 × 16.

File	PSNR (dB)			
	Gaussian	BPBD	DBBD	DPCI
barb.tiff	22.97	23.19	23.90	23.03
boat.tiff	24.73	25.21	26.89	24.89
france.tiff	16.82	18.02	19.00	16.08
goldhill2.tiff	26.05	26.61	27.99	26.41
lena2.tiff	27.06	27.62	29.39	26.92
library.tiff	15.65	16.01	17.37	15.65
mandrill.tiff	20.08	20.42	21.02	20.00
mountain.tiff	16.74	16.84	17.81	16.19
peppers2.tiff	27.10	27.80	29.09	26.05
washsat.tiff	30.93	31.08	32.28	30.72
zelda.tiff	30.76	31.53	33.36	30.90
Average	23.53	24.03	25.28	23.35

**Table 6:** Image quality results for 32 × 32.

File	PSNR (dB)			
	Gaussian	BPBD	DBBD	DPCI
barb.tiff	22.94	23.11	23.56	22.72
boat.tiff	24.84	25.36	26.04	24.49
france.tiff	17.50	17.85	18.86	16.45
goldhill2.tiff	24.93	26.46	27.23	24.62
lena.tiff	26.95	27.77	28.49	26.12
library.tiff	15.72	15.85	17.21	14.91
mandrill.tiff	20.13	20.19	20.50	20.06
mountain.tiff	16.65	16.75	17.33	15.87
peppers2.tiff	26.36	27.85	28.19	25.07
washsat.tiff	30.82	30.89	31.87	30.72
zelda.tiff	30.78	31.48	32.71	29.84
Average	23.42	23.96	24.73	22.81

**Table 7:** Energy for operations.

Operation	Energy Consumption (nJ)
Add	0.484
Subtract	0.484
Multiply	14.036
Divide	10.648
Compare	0.484

**Table 8:** Energy consumption for matrices.

Process	Energy Per Block (μJ)			
	Gaussian	BPBD	DBBD	DPCI
Constructing 8	High	High	0	0.089
Constructing 16	High	High	0	0.369
Constructing 32	High	High	0	1.506
Sensing 8	High	0.028	0.028	0
Sensing 16	High	0.112	0.112	0
Sensing 32	High	0.446	0.446	0

## Data Availability

There were no datasets created during this study and all relevant datasets are already publicly available.
